# Factors triggering knot formation in ileosigmoid knotting

**DOI:** 10.12669/pjms.38.6.6133

**Published:** 2022

**Authors:** Ercan Korkut, Sabri Selcuk Atamanalp

**Affiliations:** 1 Prof. Ercan Korkut, Assist. MD. Department of General Surgery, Faculty of Medicine, Ataturk University, Erzurum, Turkey; 2 Prof. Sabri Selcuk Atamanalp, MD. Department of General Surgery, Faculty of Medicine, Ataturk University, Erzurum, Turkey

**Keywords:** Ileum, Sigmoid colon, Ileosigmoid knotting, Triggering factors

## Abstract

**Objectives:**

Ileosigmoid knotting (ISK) is a rare double-loop ileus form. Therefore, its physiopathology including the triggering factors, are not well investigated. We aimed to discuss the physiopathology, particularly the triggering factors in ISK.

**Methods:**

The data of 80 patients with ISK were analyzed retrospectively until June 1986 and prospectively thereafter. As control group, the data of 80 patients with bowel obstruction arising from adhesive ileus or strangulated inguinal hernia were evaluated prospectively during the last 24 months. As probable triggering factors in ISK, the role of acute diarrhea, body motions, overeating, coughing, and labor were investigated.

**Results:**

Prior to the main clinical features of ISK, acute diarrhea (17 patients, 21.3%, p<0.05), harvesting (9 patients, 11.3%, p<0.05), and overeating (8 patients, 10.0%, p<0.05) were found effective.

**Conclusion:**

Although there is not enough data on this subject in the literature, increased bowel motility, excessive body motions, and overeating appear as the triggering factors in the forming of ISK.

## INTRODUCTION

Ileosigmoid knotting (ISK) is a rare double-loop ileus form, with about a few hundred cases reported to date.^1^,^2^ Nevertheless, it is relatively common in Eastern Turkey, our practice area.^3^,^4^ We have 80 cases with ISK over a 56-year period (from June 1966 to July 2022). This is one of the largest single-center ISK series in the world.^5^ Most likely due to the rarity of the disease, the physiopathology of ISK, particularly the triggering factors, are not well investigated. There are few studies on this subject in the literature.^2^ In light of our comprehensive experience, we want to investigate the possible triggering factors in ISK.

## METHODS

The data of 80 patients with ISK were analyzed retrospectively until June 1986 (56 patients) and prospectively thereafter (24 patients). As control group, the data of consecutive 80 patients with adhesive ileus or strangulated inguinal hernia were evaluated prospectively in the last 24 months. Prior to the main clinical features of intestinal obstruction including abdominal pain, distention, and obstipation, the following criteria were noted: acute diarrhea, sudden and excessive body motions, overeating after a prolonged starvation, coughing spell, and labor. This study was approved by Ethical Committee of Ataturk University Faculty of Medicine (2017/17).

In statistical analyses SPSS v22.0 was used. Student’s t test was applied for homogenous distributions, while Chi-Square test was applied to compare qualitative variables. Statistical significance was set at p<0.05.

## RESULTS

In our 80-case ISK series, 17 patients (21.3%) demonstrated a one- to three-day interval of diarrhea prior to the main clinical features of ISK (7.5% in control group, p<0.05). Interestingly, premorbid diarrhea was more frequent in childhood ISK (in three of total seven patients, 42.9%). Of our patients, 46 (57.5%) were farmer in rural areas and nine (11.3%) had a harvesting anamnesis just before the development of ISK (2.5% in control group, p<0.05). Similarly, 64 patients (80.0%) had a fasting habit and in eight cases (10.0%), ISK was seen in postprandial period following Ramadan fasting (1.3% in control group, p<0.05). Among other evaluated factors, coughing did not demonstrate any statistically significant difference, while we had no patient with premorbid labor (Table-I).

**Table-I:** The results of the evaluated factors and the statistical analyses.

Parameter	Ileosigmoid knotting group	Control group	Statistical analysis and comment
Age (mean year)	47.5	45.8	Student’s t test, t: 0.61, p>0.05
Gender (male/female)	58/22 (2.6)	55/25 (2.2)	Chi Square test, x^2^: 0.27, p>0.05
Acute diarrhea	17/80 (21.3%)	6/80 (7.5%)	Chi Square test, x^2^: 6.14, p<0.05
Harvesting	9/80 (11.3%)	2/80 (2.5%)	Chi Square test, x^2^: 4.78, p<0.05
Overeating following fasting	8/80 (10.0%)	1/80 (1.3%)	Chi Square test, x^2^: 5.77, p<0.05
Coughing spell	2/80 (2.5%)	2/80 (2.5%)	Chi Square test, x^2^: 0.00, p>0.05
Labor	0/22 (0.0%)	0/25 (0.0%)	-

## DISCUSSION

A hypermobile terminal ileum with rarely a mobile cecum and a long sigmoid colon are the main anatomical prerequisites in the forming of ISK.^1^ However, ISK does not develop in all people-at-risk and all the time. It is clear that, even if the above-mentioned anatomical prerequisite happens, an initiator effect is required to a knot formation similar to the development of volvulus formation in sigmoid volvulus.^6^ Although bowels twist physiologically from time to time, only excessive torsions arising from physiological or pathological trigger actions may primarily explain knotting process.

Firstly, in our opinion and experience, bowel hypermotility, which is mostly seen in acute diarrhea, may be an important pathological precursor factor in the development of ISK. The presence of diarrhea anamnesis in a large amount of our patients supports this idea. Although some large series don’t give information about the relationship between diarrhea and ISK, most likely due to the retrospective nature of the reports,^7^,^8^ at least, a case report by Mba et al.^9^ supports this idea. Interestingly, in our series, premorbid diarrhea was more frequent in childhood ISK, the last which is an extremely rare pathology.^10^

Secondly, sudden and excessive body motions, as in reaping, may be another precursor factor in ISK. Hence, harvesting anamnesis was present in a good portion of our patients. Although in an article by Chalya and Mabula,^8^ 76.7% of patients were from the rural areas, they gave no information about the harvesting story. Interestingly, Alver et al^1^ reported a just-married woman, in whom the symptoms of ISK commenced abruptly during the first coitus, which may be connected with the relationship between abnormal body motions and ISK. On the other hand, Ucar et al.^11^ presented a case, in whom ISK developed following a spontaneous vaginal delivery, and they suggested a cause and effect relation between labour pain and ISK, which might be thought as a kind of abnormal body motions. Similarly, Boukhalit et al^12^ reported a case, in whom, ISK was developed just following a delivery and Abebe et al^13^ presented another ISK case, who was admitted in hospital 11 hours later a delivery, and they supported the present hypothesis. Although we have three pregnant ISK cases, we have no experience about an ISK during or following a delivery. In our opinion, some special occasions causing sudden and excessive body motions may initiate ISK by forcing the hypermobile terminal ileum to rotate around the sigmoid colon.

Thirdly, an overeating after a prolonged starvation, which was present in a certain portion of our patients, may also be a precipitating factor. Banerjee et al.^14^ reported two cases suffered from ISK just after the holy month of Ramadan. Although Machado^15^ did not give any information about the incidence of Ramadan fasting in his 280-case review study, he supports the relationship between overeating and ISK. A habit of ingestion of a high-bulk food and fluid after a prolonged interval of fasting causes a rapid transit of the bowel content into the proximal jejunum, which forces the empty hypermobile terminal ileum loops to rotate and initiates ISK.^1^

## CONCLUSIONS

In conclusion, even if strong evidence is not present in the literature, increased bowel motility, excessive body motions, and overeating look like the triggering factors in ISK. Among these, diarrhea appears like more effective in childhood ISK. It is clear that the evaluation of the large ISK series may help to clarify the physiopathology of ISK, particularly the triggering factors.

### Authors’ Contribution:

**EK:** Data collection, manuscript writing, revision of the final draft.

**SSA:** Data collection, manuscript writing, revision of the final draft.

**SSA:** Is responsible for responsible and accountable for the accuracy or integrity of the work.

## References

[1] Alver O, Oren D, Tireli M, Kayabasi B, Akdemir D (1993). Ileosigmoid knotting in Turkey:Review of 68 cases. Dis Colon Rectum.

[2] Web of Science Ileosigmoid knot.

[3] Atamanalp SS (2021). Ileosigmoid knotting:An update for Atamanalp classification. Pak J Med Sci.

[4] Atamanalp SS (2014). Treatment for ileosigmoid knotting:A single-center experience of 74 patients. Tech Coloproctol.

[5] Atamanalp SS (2018). Ileosigmoid knotting:One of the largest single-center series. Pak J Med Sci.

[6] Disci E, Atamanalp SS (2022). Factors precipitating volvulus formation in sigmoid volvulus. Ulus Travma Acil Cerrahi Derg.

[7] Ooko PB, Saruni S, Oloo M, Topazian HM, White R (2016). Ileo-sigmoid knotting:a review of 61 cases in Kenya. Pan Afr Med J.

[8] Chalya PL, Mabula JB (2015). Sigmoid volvulus and ileo-sigmoid knotting:a five-year experience at a tertiary care hospital in Tanzania. World J Emerg Surg.

[9] Mba EL, Obiano SK, Mshelia NM (2018). Compound volvulus:a case report and literature review. J Surg Case Rep.

[10] Atamanalp SS, Disci E, Peksoz R, Atamanalp RS, Tatar Atamanalp C (2022). Ileosigmoid knotting:A review of 923 cases. Pak J Med Sci.

[11] Ucar NS, Yuksel BC, Hengirmen S (2009). Did ileal knotting trigger labor or did labor cause ileal knotting?Report of a case. Surg Today.

[12] Boukhalit H, Zamani O, Jroundi L (2019). A case of ileosigmoid knotting in the postpartum period. Pan Afr Med J.

[13] Abebe E, Suga Y, Tadese M, Tesfaye T (2017). Ileosigmoid knotting in grand multipara women during labor. A rare occurrence. Ethiop Med J.

[14] Banerjee C, Mukhopadhyay M, Roy A, Kumar J, Mukherjee S, Rahman QM (2014). The unusual volvulus:A five year retrospective analysis of nine cases. Indian J Surg.

[15] Machado NO (2009). Ileosigmoid knot:a case report and literature review of 280 cases. Ann Saudi Med.

